# Robot-assisted single port radical nephrectomy and cholecystectomy: description and technical aspects

**DOI:** 10.1590/S1677-5538.IBJU.2016.0560

**Published:** 2018

**Authors:** Francisco Hidelbrando Alves Mota, Luis Felipe Sávio, Rafael Eiji Sakata, Renato Fidelis Ivanovic, Marco Antonio Nunes da Silva, Ronaldo Soares Maia, Carlo Camargo Passerotti

**Affiliations:** 1 Centro de Cirurgia Robótica do Hospital Alemão Oswaldo Cruz, São Paulo. Brasil;; 2 Divisão de Urologia, Faculdade de Medicina da Universidade de São Paulo, São Paulo, Brasil

## Abstract

**Introduction:**

Robot-Assisted Single Site Radical Nephrectomy (RASS-RN) has been reported by surgeons in Europe and United States (1–3). To our best knowledge this video presents the first RASS-RN with concomitant cholecystectomy performed in Latin America.

**Case:**

A 66 year-old renal transplant male due to chronic renal failure presented with an incidental 1.3cm nodule in the upper pole of the right kidney. In addition, symptomatic gallbladder stones were detected.

**Results:**

Patient was placed in modified flank position. Multichannel single port device was placed using Hassan's technique through a 3 cm supra-umbilical incision. Standard radical nephrectomy and cholecystectomy were made using an 8.5mm camera, two 5mm robotic arms and an assistant 5mm access. Surgery time and estimated blood loss were 208 minutes and 100mL, respectively. Patient did well and was discharged within less than 48 hours, without complications. Pathology report showed benign renomedullary tumor of interstitial cells and chronic cholecystitis.

**Discussion:**

Robotic technology improves ergonomics, gives better precision and enhances ability to approach complex surgeries. Robot-assisted Single Port aims to reduce the morbidity of multiple trocar placements while maintaining the advantages of robotic surgery (2). Limitations include the use of semi-rigid instruments providing less degree of motion and limited space leading to crash between instruments. On the other hand, it is possible to perform complex and concomitant surgeries with just one incision.

**Conclusion:**

RASS-RN seems to be safe and feasible option for selected cases. Studies should be performed to better understand the results using single port technique in Urology.

## ARTICLE INFO

**Available at: http://www.intbrazjurol.com.br/video-section/20160560-Mota_et_al**

**Int Braz J Urol. 2018; 44 (Video #3): 202-3**
